# Optimization of Photovoltaic Performance of Pb-Free Perovskite Solar Cells via Numerical Simulation

**DOI:** 10.3390/molecules28010224

**Published:** 2022-12-27

**Authors:** Ali Alsalme, Malak Faisal Altowairqi, Afnan Abdullah Alhamed, Rais Ahmad Khan

**Affiliations:** Department of Chemistry, College of Science, King Saud University, Riyadh 11451, Saudi Arabia

**Keywords:** MAGeI_3_, perovskite solar cells, electron transport layer, simulation, SCAPS-1D

## Abstract

Recently, the simulation of perovskite solar cells (PSCs) via SCAPS-1D has been widely reported. In this study, we adopted SCAPS-1D as a simulation tool for the numerical simulation of lead-free (Pb-free) PSCs. We used methyl ammonium germanium iodide (MAGeI_3_) as a light absorber, zinc oxysulphide (ZnOS) as an electron transport layer (ETL), and spiro-OMeTAD as a hole transport layer. Further, the thickness of the ZnOS, MAGeI_3_, and spiro-OMeTAD layers was optimized. The optimal thicknesses of the ZnOS, MAGeI_3_, and spiro-OMeTAD layers were found to be 100 nm, 550 nm, and 100 nm, respectively. The optimized MAGeI_3_-based PSCs exhibited excellent power conversion efficiency (PCE) of 21.62%, fill factor (FF) of 84.05%, and Jsc of 14.51 mA/cm^2^. A fantastic open circuit voltage of 1.77 V was also obtained using SCAPS-1D. We believe that these theoretically optimized parameters and conditions may help improve the experimental efficiency of MAGeI_3_-based PSCs in the future.

## 1. Introduction

Organic–inorganic hybrid perovskite materials-based solar cells have been proven to be the most efficient thin film solar cell technology [1,2,3]. The perovskite term originated from the mineral calcium titanate (CaTiO_3_) [4]. Hybrid perovskite materials such as methyl ammonium lead halide (MAPbX_3_; X = halide anion, and MA = CH_3_NH_3_^+^) possess excellent properties such as low band gap, high absorption coefficient, large charge carriers lifetime, and tunable band gap [5]. These excellent optoelectronic properties of MAPbX_3_ perovskite materials attracted the scientific community to explore their potential role in photovoltaic applications [6]. Miyasaka and a research team at Toin University of Yokohama developed dye-sensitized solar cells by introducing MAPbX_3_ as visible light sensitized [7]. They found that MAPbX_3_ has excellent optoelectronic properties, and a reasonable power conversion efficiency (PCE) of less than 4% was achieved, including excellent open circuit voltage (Voc) of 0.96 V. This work opened a new window for materials scientists to develop the new thin film solar cell technology. Thus, an improved PCE of 6.5% was reported for perovskite quantum dot solar cells by Park and a research team at Sungkyunkwan University [8]. In 2012, this interesting PCE of 6.5% was enhanced to more than 9% by Kim et al. [9] by using a novel solid-state hole transport material layer (HTL). MAPbX_3_ may be a promising light absorber material for fabricating high-performance photovoltaic devices [9]. For this reason, enormous efforts were made by various research groups to further enhance the photovoltaic efficiency and performance of perovskite solar cells (PSCs) [10,11,12,13,14]. In 2021, an excellent PCE of more than 25% was reported [15]. Although MAPbX_3-_based PSCs show excellent efficiency, they still suffer from some serious drawbacks, such as poor long-term stability, moisture sensitivity, and the presence of toxic Pb^2+^ in the MAPbX_3_ structure [16,17]. The toxicity of Pb^2+^ in the MAPbX_3_ structure is a major challenge that still needs to be overcome [18]. In this connection, the optoelectronic properties of bismuth- (Bi^3+^), tin- (Sn^2+^), antimony- (Sb^3+^), and germanium-based (Ge^2+^) perovskite or perovskite-like materials have been studied [16,19,20,21,22,23,24,25]. The reported literature showed that Bi^3+^ or Sb^3+^-based perovskite-like materials exist with the molecular formula of A_3_B_2_X_9_ (A = CH_3_NH_3_^+^, B = Bi^3+^ or Sb^3+^ and X = halide anion) and possess excellent stability in moisture [22]. Unfortunately, the wide band gap of 2.2 eV and rapid crystallization of these perovskite-like materials are major challenges for constructing high-performance PSCs [23]. Despite enormous efforts, the PCE of PSCs based on these perovskite-like materials is still less than 3% [26]. In the case of Sn^2+^-based PSCs, an excellent PCE of ~13% has been achieved [27]. Similarly, Ge^2+^-based PSCs have been developed due to the less toxic nature of Ge^2+^ [28]. Previously, a few attempts were made to create MAGeI_3_-based PSCs, but the PCE of the fabricated PSCs could not be significantly [19]. According to the available data on MAPbX_3_-based PSCs, it can be clearly understood that Miyasaka and the research team achieved a poor PCE of less than 4% [7]. However, this PCE has been improved to more than 25% by utilizing novel device architectures, fabrication techniques, different electron transport layers (ETL), and HTL [15]. The thickness of the HTL or ETL and the type of ETL or HTL also played a vital role in developing PSCs. Therefore, it is believed that strategies previously used for MAPbX_3_ or MASnX_3_-based PSCs may also be helpful for MAGeI_3_-based PSCs [11,12,13,14,15].

Numerical simulation studies using SCAPS-1D have recently received extensive attention [28,29,30]. The use of SCAPS-1D to optimize conditions may be helpful in experimentally fabricating high-performance PSCs [30]. In this connection, a Pb-free perovskite material, cesium titanium bromide (Cs_2_TiBr_6_), with an indirect band gap of 1.9 eV was used as a light absorber [31]. Samanta et al. [31] numerically simulated Cs_2_TiBr_6_-based PSCs, which exhibited a PCE of 8.51%. Further, Ahmed et al. [32] also used a numerical simulation approach and reported an improved PCE of 11.49%. Alam et al. [33] found that Cs_2_AgBiBr_6_ has a band gap of 2.05 eV, and obtained results that exhibited a poor PCE of 4.48%. In further investigations, Madan et al. [34] also fabricated tandem photovoltaic cells using cesium silver bismuth antimony bromide (Cs_2_AgBi_0.75_Sb_0.25_Br_6_) as a top cell electrode material, and reported an exciting PCE of 10.08%. Cs_2_AgBiBr_6_ was also utilized as an absorber layer by Rai et al. [35], and showed a PCE of 9.98% in their study. In other works, cesium tin halide (CsSnX_3_)-based PSC devices showed a PCE of 10.46% [36]. Cesium bismuth iodide (Cs_3_Bi_2_I_9_) has a band gap of 2.2 eV, and a Cs_3_Bi_2_I_9_-based PSCs device showed a PCE of 13.69% [37]. Additionally, a CsGeI_3-_based PSCs device demonstrated a decent PCE of 10.8% [38]. The literature discussed above shows that the simulation of PSCs via SCAPS-1D may be helpful for the scientific community to experimentally apply optimized conditions in the fabrication of PSCs.

In this work, we simulated PSCs using the MAGeI_3_ absorber layer, and the effects of temperature and thickness of the absorber layer, ETL, and HTL were optimized. In further studies, various ETL and HTL layers were used to examine the effects of ETL and HTL on the performance of MAGeI_3_-based PSCs via SCAPS-1D. The optimized studies showed that a PCE of 21.62% can be achieved for the device structure of FTO(500 nm)/ZnOS(100 nm)/MAGeI_3_(550 nm)/spiro-OMeTAD(100 nm)/Au via numerical simulation. 

## 2. Results

### 2.1. Photovoltaic Investigations

#### 2.1.1. Optimization of the Absorber Layer

**Figure 1:**
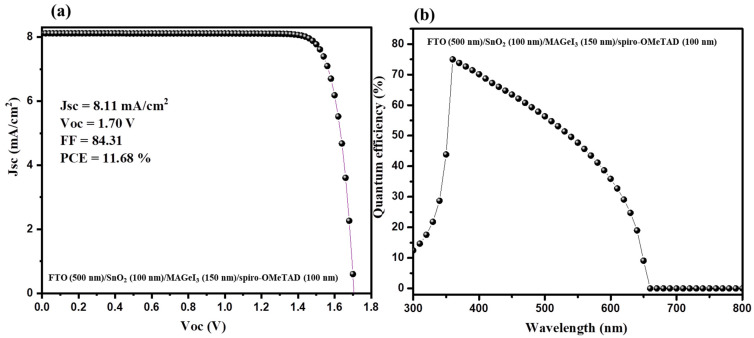
J-V curve (**a**) and EQE curve (**b**) of the simulated PSCs (FTO(500 nm)/SnO_2_(100 nm)/MAGeI_3_(150 nm)/spiro-OMeTAD(100 nm)) at 300 K.

Initially, we simulated PSCs (FTO(500 nm)/SnO_2_(100 nm)/MAGeI_3_(150 nm)/spiro-OMeTAD(100 nm)) on SCAPS-1D at the applied temperature of 300 K. The performance of these simulated PSCs was studied by obtaining their short circuit photocurrent density versus voltage (J-V) curve. The collected J-V data of these PSCs are presented in Figure 1a. The J-V results indicate an interesting PCE of 11.68% for 150 nm thick MAGeI3 layer-based PSCs at 300 K. The simulated PSCs also exhibit an excellent Voc of 1.70 V with Jsc of 8.11 mA/cm^2^. The external quantum efficiency (EQE) curve of the above simulated PSCs was also obtained, and is depicted in Figure 1b. The EQE results revealed poor quantum efficiency, which is due to the thin layer of MAGeI_3_. This quantum efficiency can be improved by increasing the thickness of the MAGeI_3_ layer.

The poor Jsc of the simulated PSCs (FTO(500 nm)/SnO_2_(100 nm)/MAGeI_3_(150 nm)/spiro-OMeTAD(100 nm)) was due to poor quantum efficiency. In some cases, temperature can influence the performance of the simulated PSCs. 

**Figure 2:**
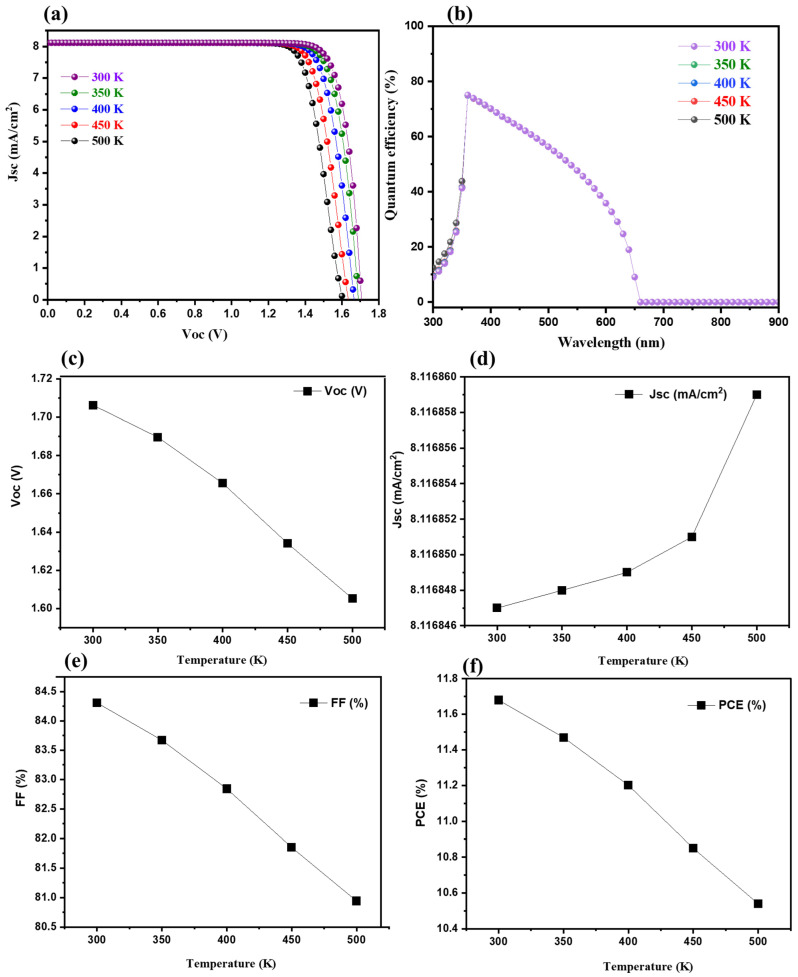
J-V curves (**a**) and EQE curves (**b**) of the simulated PSCs (FTO(500 nm)/SnO_2_(100 nm)/MAGeI_3_(150 nm)/spiro-OMeTAD(100 nm)) at different temperatures (300–500 K). Extracted Voc (**c**), Jsc (**d**), FF (**e**), and PCE (**f**) of the simulated PSCs at different temperatures (300–500 K).

**Figure 3:**
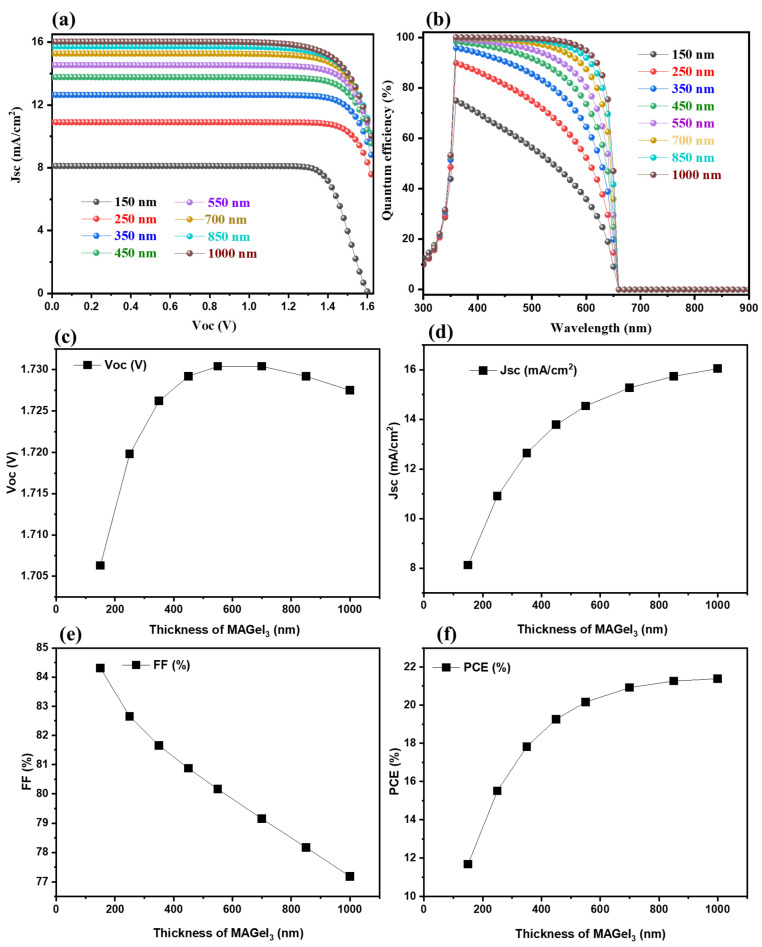
J-V curves (**a**) and EQE curves (**b**) of the simulated PSCs (FTO(500 nm)/SnO_2_(100 nm)/MAGeI_3_(150–1000 nm)/spiro-OMeTAD(100 nm)) at 300 K. Extracted Voc (**c**), Jsc (**d**), FF (**e**), and PCE (**f**) of the simulated PSCs (FTO(500 nm)/SnO_2_(100 nm)/MAGeI_3_(150–1000 nm)/spiro-OMeTAD(100 nm)) at 300 K.

In this regard, we have simulated Pb-free PSCs with a device structure of FTO(500 nm)/SnO_2_(100 nm)/MAGeI_3_(150 nm)/spiro-OMeTAD(100 nm) at different temperatures (300–500 K). The obtained J-V curves and EQE curves of the Pb-free PSCs devices are shown in Figure 2a,b, respectively. The photovoltaic parameters obtained from the J-V curves are presented in Figure 2c–f. It can be seen that an increase in temperature decreases the Voc of the simulated PSCs (Figure 2c). However, very little change in the Jsc value relating to temperature was observed (Figure 2d). The simulated EQE results (Figure 2b) also show that temperature could not alter the simulated Pb-free PSCs’ quantum efficiency and absorption properties; this is responsible for the insignificant change in the Jsc value (Figure 2c, Table S4). The fill factor (FF) and PCE of the simulated PSCs also decrease with increasing temperature, as shown in Figure 2e,f, respectively. Reported literature showed that the fabrication of low-temperature processed PSC devices is of great significance [29,30]. We also observed that the simulated Pb-free PSCs device showed a PCE of 11.68% at 300 K (Figure 2c), which is very interesting. Therefore, we have selected 300 K as the optimized temperature for further simulations. The observations showed that MAGeI_3_ might be a promising absorber layer for developing environmentally friendly PSCs. Because the thickness of the absorber layer can significantly affect the Jsc and other parameters of the simulated PSCs, we simulated further PSCs by varying the thickness of the MAGeI_3_ layer. The thickness of the MAGeI_3_ layer varied in the range from 150 nm to 1000 nm. The J-V curves of the simulated PSCs at different thicknesses of MAGeI_3_ are presented in Figure 3a, and EQE curves of the simulated PSCs at different thickness of MAGeI_3_ layer are presented in Figure 3b. Figure 3a clearly shows that the Jsc value of the simulated PSCs significantly increases with the increasing thickness of the MAGeI_3_ layer. The highest Jsc, of 16.0 mA/cm^2^, was observed for a 1000 nm thick MAGeI_3_ layer. 

The extracted photovoltaic parameters for the simulated PSCs at different thicknesses of MAGeI_3_ are presented in Figure 3c–f. The simulated results showed that the thickness of the MAGeI_3_ layer significantly affects the performance of MAGeI_3_-based PSCs. The EQE results indicated that increased MAGeI_3_ thickness improved quantum efficiency (Figure 3b). The simulated PSCs showed the maximum absorption between 350 nm and 650 nm (Figure 3b). Thus, the Jsc value of the simulated PSCs increases with the increasing thickness of the MAGeI_3_ layer (Figure 3d). This behavior may be due to the high active absorber area of the thicker MAGeI_3_ layer, which may be responsible for the generation of more photons and the improved Jsc of 16.0 mA/cm^2^ as observed. The FF value decreases with the increasing thickness of the MAGeI_3_ layer (Figure 3e). The Voc of the simulated PSCs also increases with the growing thickness of the MAGeI_3_ layer (Figure 3c). The obtained values of the photovoltaic parameters such as FF, Voc, Jsc, and PCE of the simulated PSCs related to the thickness of MAGeI_3_ are provided in Table S5. The highest PCE, of 21.39%, was obtained using a 1000 nm MAGeI_3_ layer. However, reported experimental results showed that a much thicker absorber layer may affect the recombination of charge carriers with the absorber layer [31,32]. Chen et al. [31] also reported that an absorber layer thicker than 600 nm is insensitive. Our simulated results observed that 550 nm thick MAGeI_3_ layer-based PSCs also demonstrated a good PCE of 201.16%; therefore, it would be worth using a 550 nm thick MAGeI_3_ layer for further simulation studies. For this reason, the thickness of the MAGeI_3_ layer was fixed to 550 nm for further simulation investigations.

#### 2.1.2. Selection of ETL

The PSCs consist of different components, such as light absorber layers, ETL, HTL, and metal contacts. It is necessary to match the band alignments of the different components to achieve the highest performance of the PSCs. 

**Figure 4:**
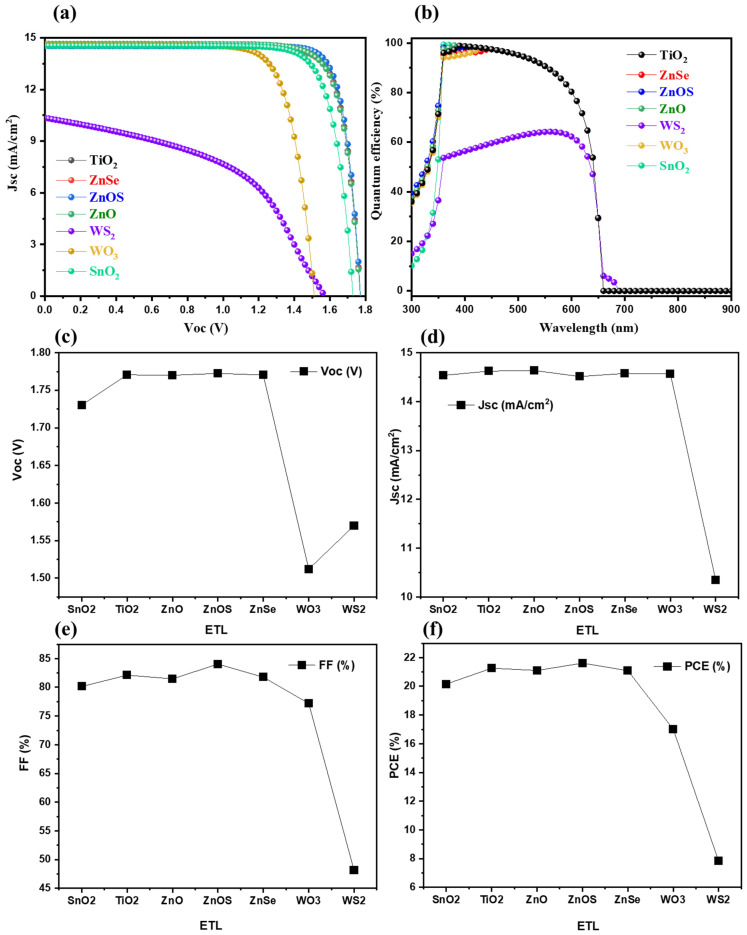
J-V curves (**a**) and EQE curves (**b**) of the simulated PSCs (FTO(500 nm)/different ETL(100 nm)/MAGeI_3_(550 nm)/spiro-OMeTAD(100 nm)) at 300 K. Extracted Voc (**c**), Jsc (**d**), FF (**e**) and PCE (**f**) of the simulated PSCs (FTO(500 nm)/different ETL(100 nm)/MAGeI_3_(550 nm)/spiro-OMeTAD(100 nm)) at 300 K.

The type of ETL or HTL used may impact the performance of the MAGeI_3_-based PSCs. ETL plays a crucial role in charge extraction and electron transportation, which are the key points for developing high-performance PSCs. Therefore, selecting a more suitable and efficient ETL is very important for better charge extraction/charge transport which may improve the efficiency of the PSCs. In this connection, we also simulated PSCs with different ETL layers (zinc oxide (ZnO), tungsten disulfide (WS_2_), titanium dioxide (TiO_2_), zinc selenide (ZnSe), zinc oxy-sulfide (ZnOS), and tungsten trioxide (WO_3_)). The above optimized conditions for the absorber layer, thickness (550 nm) and temperature (300 K), were used to further simulate the Pb-free PSCs with different ETL (100 nm) layers. The simulated J-V and EQE curves of the simulated PSCs are summarized in Figure 4a,b, respectively. The extracted photovoltaic parameters from the J-V curves are summarized in Table S6 and Figure 4c–f. WS_2_-based PSCs showed poor quantum efficiency, which suggested poor light absorption in the simulated PSCs, and the lowest PCE of 7.82% was obtained. WO_3_-based PSCs showed an improved PCE of 17.01%, and TiO_2_-based PSCs exhibited an excellent PCE of 21.27%. Meanwhile, ZnO- and ZnSe-based PSCs also showed improved PCE of 21.11 and 21.10%, respectively. This enhancement in the PCE may be due to better absorption (Figure 4b) and improved Voc values. The highest PCE of 21.62% was obtained for ZnOS-based Pb-free PSCs. This highest PCE may be attributed to the better band alignment or the electron–hole mobility of ZnOS. Therefore, we selected ZnOS as a suitable ETL for further simulation investigations. 

#### 2.1.3. Selection of HTL

Selecting a highly efficient and suitable HTL is one of the most important tasks for fabricating high-performance Pb-free PSCs. 

**Figure 5:**
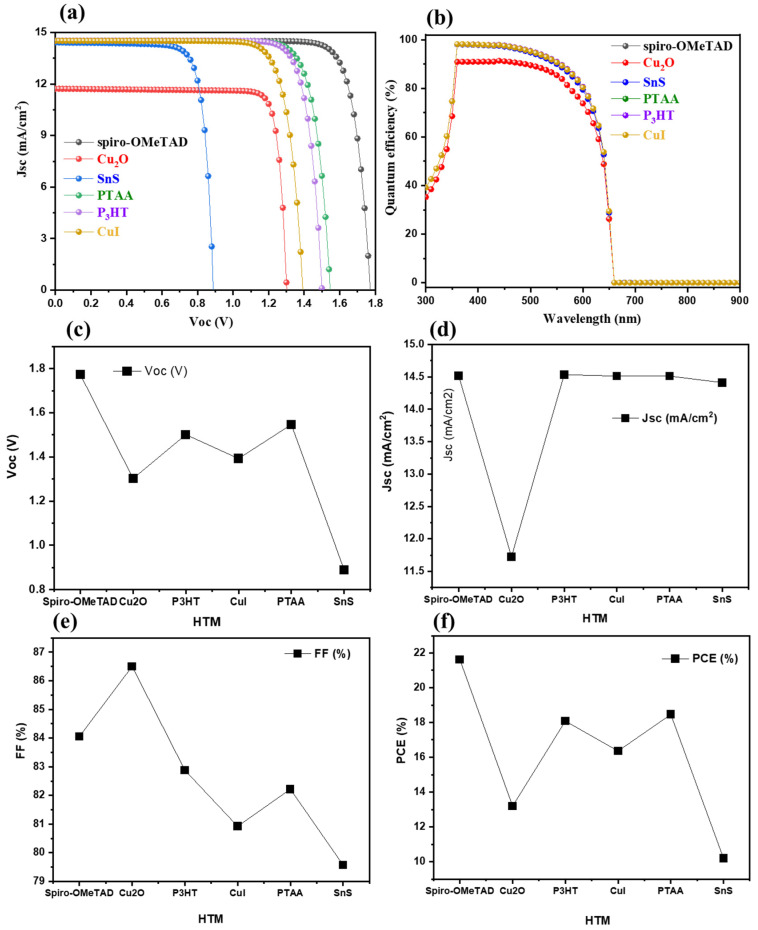
J-V curves (**a**) and EQE curves (**b**) of the simulated PSCs (FTO(500 nm)/ZnOS(100 nm)/MAGeI_3_(550 nm)/different HTL(100 nm)) at 300 K. Extracted Voc(**c**), Jsc (**d**), FF (**e**) and PCE (**f**) of the simulated PSCs (FTO(500 nm)/ZnOS(100 nm)/MAGeI_3_(550 nm)/different HTL(100 nm)) at 300 K.

In this regard, we simulated Pb-free PSCs with different HTL materials (poly(3-hexylthiophene-2,5-diyl (P_3_HT), copper oxide (Cu_2_O), copper iodide (CuI), tin sulfide (SnS), and poly(triaryl amine (PTAA)). The simulated J-V and EQE curves for the MAGeI_3_-based PSCs devices with different HTL materials are depicted in Figure 5a,b, respectively. The extracted photovoltaic parameters (Voc, Jsc, FF, and PCE) from the J-V curves are presented in Table S7 and Figure 5c–f. The J-V results indicated that a poor PCE of 10.19% was obtained for SnS HTL-based PSCs, and the highest PCE of 21.62% was obtained for spiro-OMeTAD HTL-based PSCs (Figure 5a; Table S7). On the other hand, Cu_2_O, CuI, P_3_HT, and PTAA HTL-based Pb-free PSCs exhibited PCE of 13.20%, 16.36%, 18.08%, and 18.46%, respectively. The results suggested that spiro-OMeTAD is the most suitable HTL layer compared with the other HTL layers. The highest PCE of 21.62% can be achieved for spiro-OMeTAD HTL-based PSCs.

#### 2.1.4. Effect of Thickness of ZnOS Layer

**Figure 6:**
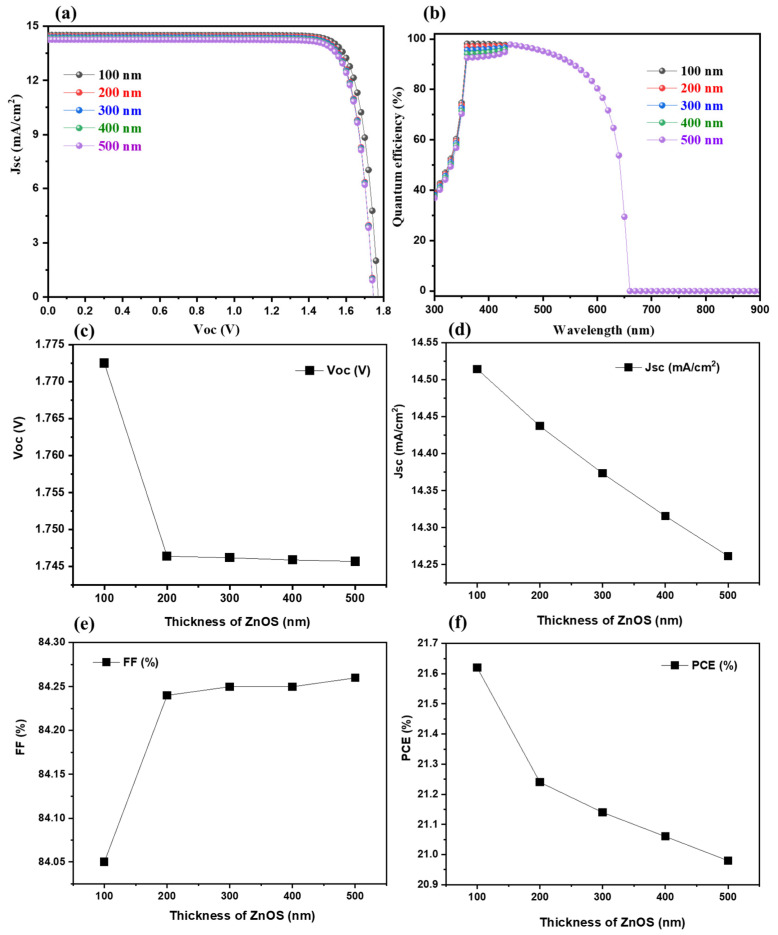
J-V curves (**a**) and EQE curves (**b**) of the simulated PSCs (FTO(500 nm)/ZnOS(100–500 nm)/MAGeI_3_(550 nm)/spiro-OMeTAD(100 nm)) at 300 K. Extracted Voc (**c**), Jsc (**d**), FF (**e**) and PCE (**f**) of the simulated PSCs (FTO(500 nm)/ZnOS(100–500 nm)/MAGeI_3_(550 nm)/spiro-OMeTAD(100 nm)) at 300 K.

The thickness of the ZnOS layer may significantly affect the photovoltaic performance of Pb-free PSCs. Hence, we investigated the influence of various thicknesses of ZnOS layer on the photovoltaic performance of the MAGeI_3_-based PSCs. The obtained J-V curves of the simulated PSCs at various ZnOS layer thicknesses are presented in Figure 6a, and EQE curves of the simulated PSCs at various thicknesses of ZnOS are shown in Figure 6b. The extracted Voc, Jsc, FF, and PCE from the J-V curves of the simulated PSCs are shown in Figure 6c–f, respectively. The simulation studies revealed that the simulated PSCs’ Voc, Jsc, and PCE decreased with increasing ZnOS layer thickness (Figure 6a and Table S8). This indicates that a thin ZnOS layer may provide better charge extraction and transportation. Therefore, a 100 nm thick ZnOS layer would be more promising and efficient for fabricating high-performance PSCs.

#### 2.1.5. Effect of Thickness of the Spiro-OMeTAD Layer

**Figure 7:**
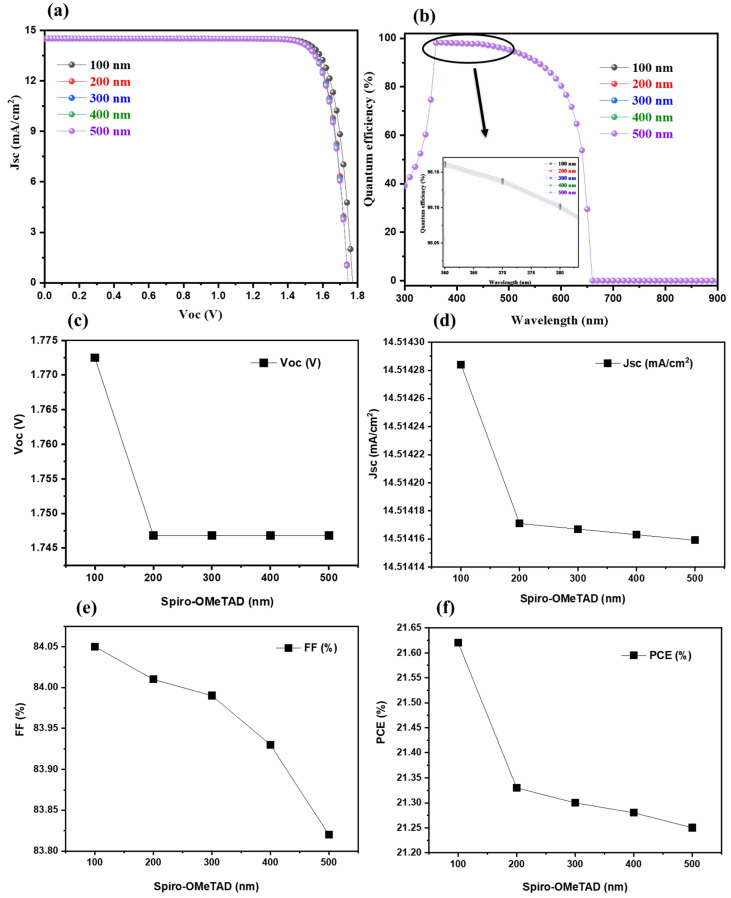
J-V curves (**a**), EQE curves (**b**) of the simulated PSCs (FTO(500 nm)/ZnOS(100 nm)/MAGeI_3_(550 nm)/spiro-OMeTAD(100–500 nm)) at 300 K. Inset of Figure 7b shows enlarged view of EQE curve. Extracted Voc (**c**), Jsc (**d**), FF (**e**), and PCE (**f**) of the simulated PSCs (FTO(500 nm)/ZnOS(100 nm)/MAGeI_3_(550 nm)/spiro-OMeTAD(100–500 nm)) at 300 K.

**Figure 8:**
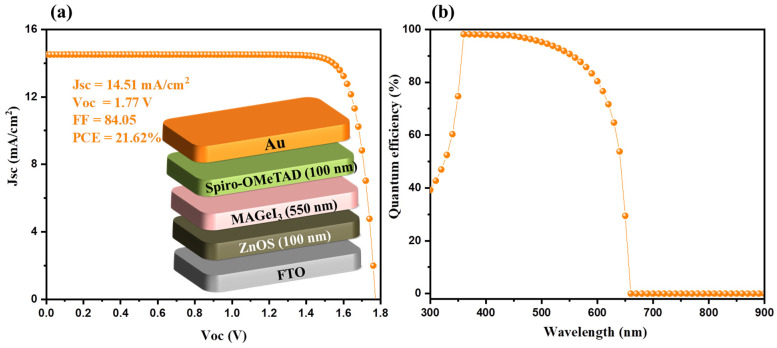
J-V curve (**a**) and EQE curve (**b**) of the optimized, best-performing Pb-free PSCs (FTO (500nm)/ZnOS(100 nm)/MAGeI_3_(550 nm)/spiro-OMeTAD(100 nm)). The inset of Figure 1a shows the optimized device structure.

Lastly, we also investigated the influence of various thicknesses of the spiro-OMeTAD layer on the photovoltaic performance of the MAGeI_3_-based PSCs. The thickness of the spiro-OMeTAD layer varied from 100 nm to 500 nm. The obtained J-V and EQE curves of the simulated Pb-free PSCs at different thicknesses of spiro-OMeTAD layer are presented in Figure 7a,b, respectively. The extracted J-V parameters from the J-V curves are summarized in Figure 7c–f and Table S9. From the collected J-V graphs of the simulated PSCs, it can be seen that the Voc, Jsc, FF, and PCE of the simulated PSCs devices decrease with increasing thickness of the spiro-OMeTAD layer from 100 nm to 500 nm (Table S9). These results indicated that a 100 nm thick spiro-OMeTAD layer is more efficient for developing MAGeI_3_-based Pb-free PSCs. The J-V and EQE curve of the optimized best-performing PSCs are displayed in Figure 8a,b, respectively. The EQE curve of the optimized PSCs showed the highest quantum efficiency and the highest absorption of 80 to 98%, seen in the wavelength region of 350 nm to 600 nm (Figure 8b). Thus, the best-performing simulated PSCs device exhibited the highest PCE of 21.62% with the optimized device architecture of FTO(500 nm)/ZnOS(100 nm)/MAGeI_3_(550 nm)/spiro-OMeTAD(100 nm). To further validate our simulation calculation and methods, we numerically simulated PSCs for the reported device (TiO_2_/MASnI_3_/spiro-OMeTAD) [39]. The simulated results are presented in Figure S2. In the reference [39], FF of 67.19%, PCE of 16.71%, Jsc of 26.90 mA/cm^2^, and Voc of 0.924 V were reported for TiO_2_/MASnI_3_/spiro-OMeTAD PSCs. In our simulations, FF of 67.49%, PCE of 16.6%, Jsc of 26.87 mA/cm^2^, and Voc of 0.915 V are obtained for TiO_2_/MASnI_3_/spiro-OMeTAD PSCs. 

The obtained results are consistent with the reference report [39]. This validated our employed simulation model and method. We compared our simulated results with recently reported literature. Our simulated results using MAGeI_3_ as an absorber layer are comparable with previous studies (Table 1). 

## 3. Materials and Methods

### Device Simulation

**Scheme 1:**
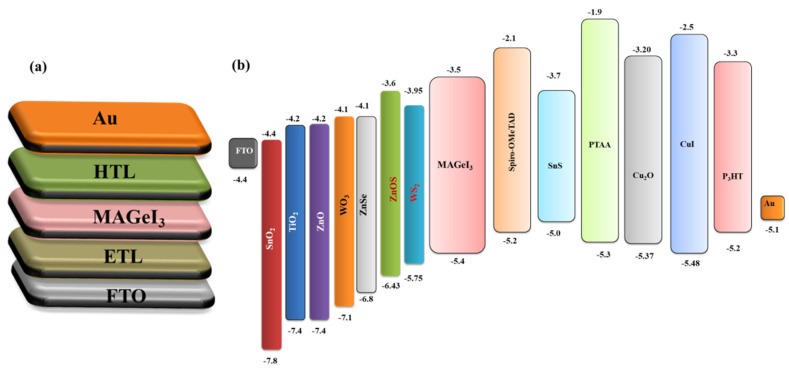
Device structure (**a**) and energy level diagram (**b**) of MAGeI_3_-based PSCs. Energy values were taken from the reported literature [31,32,33,34,35,36,37,38,40].

The MAGeI_3_-based PSCs device was simulated using SCAPS-1D software. The SCAPS-1D software was introduced by Prof. Marc Burgelman [40]. The schematic diagram of the simulated PSCs is depicted in Scheme 1a. The energy level values and diagram for the different components of PSCs are shown in Scheme 1b. 

The SCAPS-1D software worked on the principle of Poisson and continuity equations. The Poisson and continuity equations are given below [31,32,35]:

∇^2^ψ = q/ε (n-p + NA-ND) (1)

Herein, ND = donor concentration, NA = acceptor concentration, and ψ = electrostatic potential. 

The continuity equations are provided below:

∇.Jn − q ∂n/∂t = +qR(2)

∇.Jp + q ∂p/∂t = −qR(3)

In the above equations, Jp = holes current density, Jn = electrons current density, and R = carrier recombination rate.

The Drift–Diffusion Current Relations can be illustrated by the equations given below:

Jn = qnµnE + qDn ∇n(4)

Jp = qpµpE − qDp ∇p(5)

where Dn = electron diffusion coefficient and Dp = hole diffusion coefficient. 

The MAGeI_3_-based PSCs devices were simulated using SCAPS-1D software with an applied illumination of AM 1.5 G (100 mW/cm^2^; temperature range = 300–500 K). The values such as dielectric permittivity, band gap, electron affinity, etc., for MAGeI_3_, ETLs, and HTLs were taken from the reported literature. These values are presented in Tables S1–S3. The flow chart for the simulation process is illustrated in Figure S1 in Supplementary Materials. 

## 4. Conclusions

In summary, the numerical simulation study of MAGeI_3_-based PSCs was carried out using solar cell capacitance simulation (SCAPS-1D) software. The PSCs with device architecture of FTO/ZnOS/MAGeI_3_/spiro-OMeTAD/Au were numerically simulated using SCAPS-1D. Further, simulation temperature was also optimized, and the simulated PSCs exhibited improved performance at 300 K. Subsequently, various ETL layers such as ZnOS, SnO_2_, TiO_2_, ZnSe, WO_3,_ and WS_2_ were also used for further simulation studies. Simulated results suggested that ZnOS is a more suitable ETL than SnO_2_, TiO_2_, ZnSe, WO_3,_ or WS_2_. Various HTL layers such as P_3_HT, CuI, SnS, PTAA, spiro-OMeTAD, and Cu_2_O were also adopted for further simulation studies. Spiro-OMeTAD-based PSCs showed improved performance compared with other HTL layers-based PSCs. The highest PCE, of 21.62%, was achieved for the optimized PSCs.

## Figures and Tables

**Table 1:** Comparison of the efficiency of MAGeI_3_-based PSCs with recent reports [31,32,33,34,35,36,37,38].

Absorber	Voc(V)	FF (%)	Jsc (mA/cm^2^)	PCE (%)	Eg (eV)	Thickness (nm)	References
MAGeI_3_	1.77	84.05	14.51	21.62	1.9	550	This study
Cs_2_TiBr_6_	1.12	73.59	10.25	8.51	1.9	330	[31]
Cs_2_TiBr_6_	1.53	86.45	8.66	11.49	1.8	200	[32]
Cs_2_AgBiBr_6_	0.91	44.02	11.10	4.48	2.05	500	[33]
Cs_2_AgBi_0.75_Sb_0.25_Br_6_	1.14	58.5	15.1	10.08	1.8	380	[34]
Cs_2_AgBiBr_6_	0.99	66.88	14.51	9.98	2.05	-	[35]
CsSnCl_3_	0.87	56.00	19.82	9.66	1.85	-	[36]
CsSnBr_3_	0.85	58.00	21.23	10.46	1.75	-	[36]
Cs_3_Bi_2_I_9_	0.92	68.05	21.91	13.69	2.2	1500	[37]
CsGeI_3_	0.66	73.49	22.08	10.8	1.36	-	[38]

## Supplementary Materials

The following supporting information can be downloaded at: https://www.mdpi.com/article/10.3390/molecules28010224/s1, Table S1: Numerical parameters [28,41,42], Table S2: Numerical parameters [28,42,43,44,45], Table S3: Numerical parameters [28,42,43,44,45,46,47,48], Table S4: Photovoltaic parameters, Table S5: Photovoltaic parameters, Table S6: Photovoltaic parameters, Table S6: Photovoltaic parameters, Table S7: Photovoltaic parameters, Table S8: Photovoltaic parameters, Table S9: Photovoltaic parameters, Figure S1: Flow chart, Figure S2: J-V curve [39].
