# Supplementary material for: Optimization of Photovoltaic Performance of Pb-Free Perovskite Solar Cells via Numerical Simulation
Source: Molecules. 2022 Dec 27;28(1):224. doi: 10.3390/molecules28010224 (PMC9822055; doi:10.3390/molecules28010224)
Supplement: Supplementary file 1 [file molecules-28-00224-s001.zip › molecules-2089981-supplementary.pdf]

Supporting Information

Optimization of Photovoltaic Performance of Pb-Free Perovskite Solar Cells via Numerical Simulation

Ali Alsalme *, Malak Faisal Altowairqi, Afnan Abdullah Alhamed and Rais Ahmad Khan

Department of Chemistry, College of Science, King Saud University, Riyadh 11451, Saudi Arabia
* Correspondence: aalsalme@ksu.edu.sa

Table S1. Parameters used for numerical simulation [1-3].

**Parameters**	**FTO**	**SnO_2**	**$MAGeI_3$**	**Spiro-OMeTAD**
Thickness (nm)	500 nm	100 nm	150-1000 nm	100-500 nm
Band Gap (eV)	3.4	3.4	1.9	3.1
Electron affinity (eV)	4.4	4.4	3.5	2.1
Dielectric permittivity	9	9	10	3
CB effective density of states (1 cm^{-3})	2.2 x 10^{18}	2.2 x 10^{18}	1 x 10^{16}	2.2 x 10^{18}
VB effective density of states (1 cm^{-3})	1.8 x 10^{19}	1.8 x 10^{19}	1 x 10^{15}	1.8 x 10^{19}
Electron thermal velocity (cm S^{-1})	1 x 10^7	1 x 10^7	1 x 10^7	1 x 10^7
Hole thermal velocity (cm S^{-1})	1 x 10^7	1 x 10^7	1 x 10^7	1 x 10^7
Electron mobility (cm^2 VS^{-1})	20	20	16.2	2 x 10^{-4}
Hole mobility (cm^2 VS^{-1})	10	10	10.1	2 x 10^{-4}
Shallow uniform donor density N_D (1 cm^{-3})	2 x 10^{19}	2 x 10^{19}	1 x 10^9	0
Shallow uniform acceptor density N_A (1 cm^{-3})	-	0	1 x 10^9	2 x 10^{18}

Defect density N_t (1 cm^{-3})	1 x 10^{15}	1 x 10^{15}	1 x 10^{14}	1 x 10^{15}

Table S2. Numerical parameters used for different ETL [2-6].

**Parameters**	**TiO_2**	**WS_2**	**ZnO**	**ZnOS**	**ZnSe**	**WO_3**
Thickness (nm)	100 nm	100 nm	100 nm	100-500 nm	100 nm	100 nm
Band Gap (eV)	3.2	1.8	3.2	2.83	2.7	3
Electron affinity (eV)	4.2	3.95	4.2	3.6	4.1	4.1
Dielectric permittivity	10	13.60	9	9	8.6	5.76
CB effective density of states (1 cm^{-3})	2.2 x 10^{18}	1 x 10^{18}	3.7 x 10^{18}	2.2 x 10^{18}	2.2 x 10^{18}	1.96 x 10^{19}
VB effective density of states (1 cm^{-3})	1.8 x 10^{19}	1 x 10^{18}	1.8 x 10^{19}	1.8 x 10^{19}	1.8 x 10^{19}	1.96 x 10^{19}
Electron thermal velocity (cm S^{-1})	1 x 10^{7}	1 x 10^{7}	1 x 10^{7}	1 x 10^{7}	1 x 10^{7}	1 x 10^{7}
Hole thermal velocity (cm S^{-1})	1 x 10^{7}	1 x 10^{7}	1 x 10^{7}	1 x 10^{7}	1 x 10^{7}	1 x 10^{7}
Electron mobility (cm^2 VS^{-1})	100	50	100	100	110	10
Hole mobility (cm^2 VS^{-1})	25	50	25	25	400	10
Shallow uniform donor density N_D (1 cm^{-3})	1 x 10^{19}	1 x 10^{18}	5 x 10^{17}	2 x 10^{18}	1 x 10^{18}	3.68 x 10^{19}
Shallow uniform acceptor density N_A (1 cm^{-3})	0	0	0	0	0	0
Defect density N_t (1 cm^{-3})	1 x 10^{15}	1 x 10^{15}	1 x 10^{15}	1 x 10^{15}	1 x 10^{15}	1 x 10^{15}

Table S3. Numerical parameters used for different HTL [2-9].

**Parameters**	**P_3HT**	**Cu_2O**	**CuI**	**PTAA**	**SnS**
Thickness (nm)	100	100	100	100	100
Band Gap (eV)	2	2.17	2.98	2.95	1.6
Electron affinity (eV)	3.2	3.20	2.1	2.3	4.1
Dielectric permittivity	3	7.1	6.5	3.5	13
CB effective density of states (1 cm^{-3})	2.5 x 10^{18}	2.02 x 10^{17}	2.8 x 10^{19}	2.2 x 10^{18}	1.18 x 10^{18}
VB effective density of states (1 cm^{-3})	1.8 x 10^{19}	1.0 x 10^{19}	1.0 x 10^{19}	1.8 x 10^{19}	4.46 x 10^{18}
Electron thermal velocity (cm S^{-1})	1 x 10^7	1 x 10^7	1 x 10^7	1 x 10^7	1 x 10^7
Hole thermal velocity (cm S^{-1})	1 x 10^7	1 x 10^7	1 x 10^7	1 x 10^7	1 x 10^7
Electron mobility (cm^2 VS^{-1})	1 x 10^{-4}	200	1.69 x 10^{-4}	1 x 10^{-4}	15
Hole mobility (cm^2 VS^{-1})	1 x 10^{-4}	80	1.69 x 10^{-4}	1 x 10^{-4}	100
Shallow uniform donor density N_D (1 cm^{-3})	2 x 10^{18}	2 x 10^{18}	0	**0**	0
Shallow uniform acceptor density N_A (1 cm^{-3})	0	0	1 x 10^{18}	1 x 10^{18}	1.1 x 10^{16}
Defect density N_t (1 cm^{-3})	1 x 10^{14}	1 x 10^{14}	1 x 10^{15}	1 x 10^{15}	1 x 10^{15}

Table S4. Photovoltaic parameters of FTO/SnO_2(100 nm)/$MAGeI_3$(150 nm)/spiro-OMeTAD(100 nm) at different temperatures (300-500 K).

**Thickness of $MAGeI_3$ (nm)**	**Temperature (K)**	**Thickness of spiro-OMeTAD (nm)**	**Thickness of SnO_2 (nm)**	**Thickness of FTO (nm)**	**Voc (V)**	**Jsc (mA/cm^2)**	**FF (%)**	**PCE (%)**
150	300	100	100	500	1.6052	8.114923	80.94	10.54
150	325	100	100	500	1.6341	8.114913	81.85	10.85
150	350	100	100	500	1.6655	8.115252	82.84	11.20
150	375	100	100	500	1.6896	8.116031	83.67	11.47
150	400	100	100	500	1.7063	8.116847	84.31	11.68
150	425	100	100	500	1.7173	8.117371	84.68	11.80
150	450	100	100	500	1.7248	8.117598	84.73	11.86
150	475	100	100	500	1.7300	8.117639	84.48	11.86
150	500	100	100	500	1.7335	8.117598	84.06	11.83

Table S5. Photovoltaic parameters of FTO/SnO_2(100 nm)/$MAGeI_3$(150-1000 nm)/spiro-OMeTAD(100 nm) at temperature of 400 K.

**Thickness of $MAGeI_3$ (nm)**	**Temperature (K)**	**Thickness of spiro-OMeTAD (nm)**	**Thickness of SnO_2 (nm)**	**Thickness of FTO (nm)**	**Voc (V)**	**Jsc (mA/cm^2)**	**FF (%)**	**PCE (%)**
150	400	100	100	500	1.7063	8.116847	84.31	11.68
250	400	100	100	500	1.7198	10.903281	82.65	15.50
350	400	100	100	500	1.7262	12.645160	81.65	17.82
450	400	100	100	500	1.7292	13.775897	80.87	19.26
550	400	100	100	500	1.7304	14.537314	80.16	20.16
700	400	100	100	500	1.7304	15.274751	79.15	20.92
850	400	100	100	500	1.7292	15.735923	78.17	21.27
1000	400	100	100	500	1.7275	16.043349	77.18	21.39

Table S6. Photovoltaic parameters of FTO/different ETL(100 nm)/$MAGeI_3$(550 nm)/spiro-OMeTAD(100 nm) at temperature of 400 K.

Thickness of $MAGeI_3$ (nm)	Temperature (K)	Thickness of spiro-OMeTAD (nm)	ETL (thickness=100 nm)	Thickness of FTO (nm)	Voc (V)	Jsc (mA/cm^2)	FF (%)	PCE (%)
550	400	100	SnO_2	500	1.7304	14.537314	80.16	20.16
550	400	100	TiO_2	500	1.7707	14.621755	82.14	21.27
550	400	100	ZnO	500	1.7700	14.637237	81.49	21.11
550	400	100	ZnOS	500	1.7725	14.514284	84.05	21.62
550	400	100	ZnSe	500	1.7704	14.576617	81.78	21.10
550	400	100	WO_3	500	1.5122	14.570763	77.19	17.01
550	400	100	WS_2	500	1.5700	10.349160	48.13	7.82

Table S7. Photovoltaic parameters of FTO/ZnOS(100 nm)/$MAGeI_3$(550 nm)/different HTL(100 nm) at temperature of 400 K.

Thickness of $MAGeI_3$ (nm)	Temperature (K)	HTM (thickness=100 nm)	Thickness of ZnOS (nm)	Thickness of FTO (nm)	Voc (V)	Jsc (mA/cm^2)	FF (%)	PCE (%)
550	400	Spiro-OMeTAD	100	500	1.7725	14.514284	84.05	21.62
550	400	Cu_2O	100	500	1.3017	11.724010	86.50	13.20
550	400	P_3HT	100	500	1.5006	14.534840	82.88	18.08
550	400	CuI	100	500	1.3926	14.513732	80.92	16.36
550	400	PTAA	100	500	1.5468	14.513927	82.22	18.46
550	400	SnS	100	500	0.8890	14.411546	79.57	10.19

Table S8. Photovoltaic parameters of the FTO/ZnOS(100-500 nm)/$MAGeI_3$(550 nm)/spiro-OMeTAD(100 nm) at temperature of 400 K.

**Thickness of $MAGeI_3$ (nm)**	**Temperature (K)**	**Thickness of Spiro-OMeTAD (nm)**	**Thickness of ZnOS (nm)**	**Thickness of FTO (nm)**	**Voc (V)**	**Jsc (mA/cm^2)**	**FF (%)**	**PCE (%)**
550	400	100	100	500	1.7725	14.514284	84.05	21.62
550	400	100	200	500	1.7464	14.437178	84.24	21.24
550	400	100	300	500	1.7462	14.373313	84.25	21.14
550	400	100	400	500	1.7459	14.315627	84.25	21.06
550	400	100	500	500	1.7457	14.261470	84.26	20.98

Table S9. Photovoltaic parameters of the FTO/ZnOS(100 nm)/$MAGeI_3$(550 nm)/spiro-OMeTAD(100-500 nm) at temperature of 400 K.

**Thickness of $MAGeI_3$ (nm)**	**Temperature (K)**	**Thickness of Spiro-OMeTAD (nm)**	**Thickness of ZnOS (nm)**	**Thickness of FTO (nm)**	**Voc (V)**	**Jsc (mA/cm^2)**	**FF (%)**	**PCE (%)**
550	400	100	100	500	1.7725	14.514284	84.05	21.62
550	400	200	100	500	1.7468	14.514171	84.01	21.33
550	400	300	100	500	1.7468	14.514167	83.99	21.30
550	400	400	100	500	1.7468	14.514163	83.93	21.28
550	400	500	100	500	1.7468	14.514159	83.82	21.25

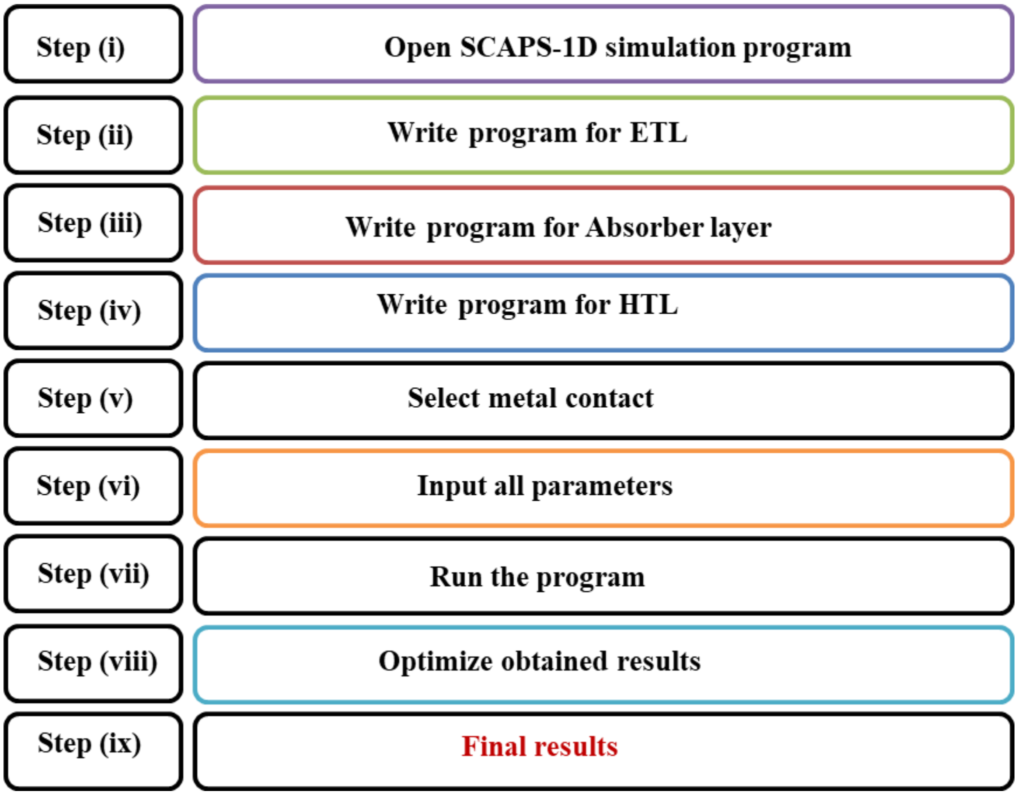

Figure S1. Flow chart for the simulation of PSCs on SCAP-1D.

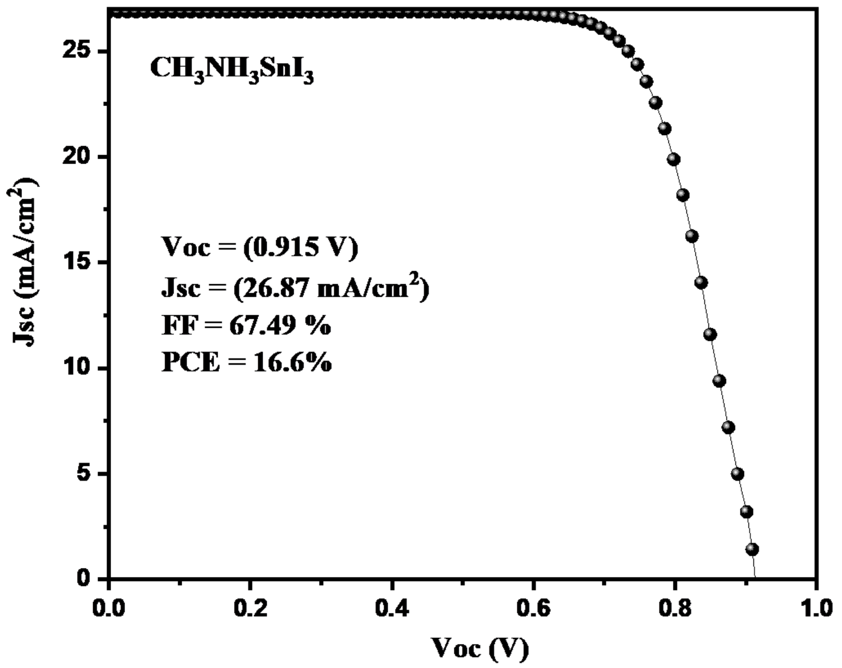

Figure S2. J-V curve for the simulated reference device [10].

References

[1] A. Tara, V. Bharti, S. Sharma, R. Gupta, Device simulation of $FASnI_3$ based perovskite solar cell with $Zn(O_{0.3}, S_{0.7})$ as electron transport layer using SCAPS-1D, Opt Mater. 119 (2021) 111362.

[2] M.A. Rahman, Design and simulation of a high-performance Cd-free Cu_2SnSe_3 solar cells with SnS electron-blocking hole transport layer and TiO_2 electron transport layer by SCAPS-1D. SN Appl. Sci. 3, (2021) 253.

[3] N. Lakhdar, A. Hima, Electron transport material effect on performance of perovskite solar cells based on $CH_3NH_3GeI_3$, Opt. Mater. 99 (2020) 109517.

[4] Y. Raoui, H. Ez-Zahraouy, S. Ahmad, S. Kazim, Unravelling the theoretical window to fabricate high performance inorganic perovskite solar cells, Sustainable Energy Fuels, 5 (2021) 219–229.

[5] M. K. Otoufi, M. Ranjbar, A. Kermanpur, N. Taghavinia, M. Minbashi, M. Forouzandeh, F. Ebadi, Enhanced performance of planar perovskite solar cells using TiO_2/SnO_2 and TiO_2/WO_3 bilayer structures: Roles of the interfacial layers, Sol. Energ. 208 (2020) 697-707.

[6] N. Singh, A. Agarwal, M. Agarwal, Performance evaluation of lead–free double-perovskite solar cell, Opt. Mater. 114 (2021) 110964.

[7] A. Mohandes, M. Moradi, H. Nadgaran, Numerical simulation of inorganic $Cs_2AgBiBr_6$ as a lead-free perovskite using device simulation SCAPS-1D. Opt. Quant. Electron. 53 (2021) 319.

[8] S. Z. Haider, H. Anwar. M. Wang, A comprehensive device modelling of perovskite solar cell with inorganic copper iodide as hole transport material, Semicond. Sci. Technol. 33 (2018) 035001.

[9] M.I. Hossain, F.H. Alharbi, N. Tabet, Copper oxide as inorganic hole transport material for lead halide perovskite based solar cells, Sol. Energy 120 (2015) 370–380.

[10] A.K. Singh, S. Srivastava, A. Mahapatra, J.K. Baral, B.Pradhan, Performance optimization of lead free-$MASnI_3$ based solar cell with 27% efficiency by numerical simulation, Optical Materials, 117 (2021) 111193.
